# Multi-Elemental Composition Data Handled by Chemometrics for the Discrimination of High-Value Italian Pecorino Cheeses

**DOI:** 10.3390/molecules26226875

**Published:** 2021-11-15

**Authors:** Francesca Di Donato, Martina Foschi, Nadia Vlad, Alessandra Biancolillo, Leucio Rossi, Angelo Antonio D’Archivio

**Affiliations:** Dipartimento di Scienze Fisiche e Chimiche, Università degli Studi dell’Aquila, Via Vetoio, 67010 L’Aquila, Italy; martina.foschi@univaq.it (M.F.); nadiavlad@outlook.it (N.V.); leucio.rossi@univaq.it (L.R.); angeloantonio.darchivio@univaq.it (A.A.D.)

**Keywords:** Pecorino cheese, geographical origin, multi-elemental composition, ICP-OES, PLS-DA

## Abstract

The multi-elemental composition of three typical Italian Pecorino cheeses, Protected Designation of Origin (PDO) Pecorino Romano (PR), PDO Pecorino Sardo (PS) and Pecorino di Farindola (PF), was determined by Inductively Coupled Plasma Optical Emission Spectrometry (ICP-OES). The ICP-OES method here developed allowed the accurate and precise determination of eight major elements (Ba, Ca, Fe, K, Mg, Na, P, and Zn). The ICP-OES data acquired from 17 PR, 20 PS, and 16 PF samples were processed by unsupervised (Principal Component Analysis, PCA) and supervised (Partial Least Square-Discriminant Analysis, PLS-DA) multivariate methods. PCA revealed a relatively high variability of the multi-elemental composition within the samples of a given variety, and a fairly good separation of the Pecorino cheeses according to the geographical origin. Concerning the supervised classification, PLS-DA has allowed obtaining excellent results, both in calibration (in cross-validation) and in validation (on the external test set). In fact, the model led to a cross-validated total accuracy of 93.3% and a predictive accuracy of 91.3%, corresponding to 2 (over 23) misclassified test samples, indicating the adequacy of the model in discriminating Pecorino cheese in accordance with its origin.

## 1. Introduction

Pecorino cheese is an Italian dairy product obtained from raw or thermized ewes’ whole milk. Milk coagulation, promoted by different types of rennet, is sometimes followed by a cooking step. After whey drainage and salting, Pecorino cheese is ripened for a variable time, in which lipolytic, proteolytic and glycolytic processes generate a variety of chemical substances responsible for the appreciated taste and aroma [1]. According to the ripening time, Pecorino cheeses are generally classified as *dolce* (soft-ripening), *maturo* (hard-ripening) or *da grattugia* (for grating). The final peculiar taste and flavour is closely related to the cheesemaking conditions, which often reflect the local or regional know-how (kind of starters and coagulants, curd heating temperature and ripening time) adopted in the production territory. This factor, together with the origin of milk, confers to Pecorino cheese a strong geographical identity, certified in several cases by specific EU marks [2], such as Protected Designation of Origin (PDO), Protected Geographical Indication (PGI) and Traditional Speciality Guaranteed (TSG). 

The certified dairy products, on one side, present a higher market value than other similar products but, on the other, are more susceptible to frauds. Partial of total substitution of authentic material with cheaper components (replacement of the premium milk with cheaper one, for instance) and addition of non-milk fat/oil or adulterants are among the fraudulent practices aimed at maximising the commercial profit [3]. False labelling concerning the geographical origin or substitution of raw materials coming from the certified area with similar products from other regions/countries is also an illegal practice, which is more difficult to uncover by application of conventional analytical methods because of the lack of specific markers related to the production site. The issue of geographical traceability and authentication of dairy products has been faced by application of various instrumental methods [4,5] followed by processing of the data with the use of unsupervised and supervised pattern recognition methods.

Compared to the profiling of molecular species or spectroscopic fingerprint methods, the determination of mineral and trace elements offers several advantages in food authentication and traceability [6]: (i) the multi-elemental composition closely reflects the composition of the soil or the environment in which foodstuffs are produced, (ii) it is stable and not substantially altered in the sample preparation, (iii) a relatively simple digestion process is required as pre-analytical step, and (iv) several elements can by simultaneously determined by atomic spectroscopy or spectrometry on plasmon atomizers. In view of the above advantages, profiles of mineral and trace elements have been successfully utilised for the geographical discrimination of a wide variety of foodstuffs [7,8,9,10,11,12] but have been rarely used as authenticity or geographical markers of milk and dairy products [4]. Multi-elemental composition, in particular, was considered, alone or in combination with other instrumental responses, to discriminate milk produced in Malaysia [13] or in Southern Italy [14] from milk coming from other countries. The most relevant investigation concerning cheeses, based on the combination of trace elements and isotopic ratios of biogenic elements (H, C, N and S), was aimed at differentiating PDO Parmigiano Reggiano from European and extra-European imitators, and predict the origin of seven types of European hard cheeses [15]. Combination of isotopic and elemental profiles were also used to attempt the authentication of Mozzarella di Bufala Campana, a mozzarella cheese obtained from whole buffalo milk and certified by PDO mark [16,17].

**Figure 1:**
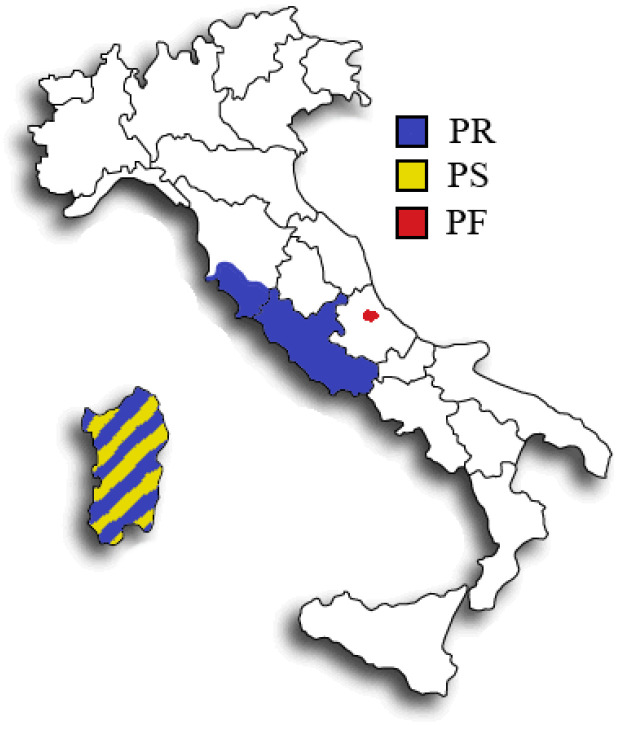
Geographical origin of Pecorino Romano (PR), Pecorino Sardo (PS), and Pecorino di Farindola (PF).

In this work, we evaluated the potentiality of multi-elemental composition determined by Inductively Coupled Plasma Optical Emission Spectrometry (ICP-OES) in the discrimination of three typical high added-value Italian Pecorino cheeses: two PDO specialties [2], namely Pecorino Romano (PR) and Pecorino Sardo (PS), and Pecorino di Farindola (PF), included by Slow Food Foundation for Biodiversity in the list of traditional food to safeguard [18]. According to the PDO specifications, milk and any other raw material originate from Sardinia in the case of PS, and from Sardinia, Lazio and Province of Grosseto (Tuscany) in the case of PR (Figure 1). PF is produced in a restricted mountain area within the National Park of Gran Sasso and Monti della Laga located in the central Apennines (Abruzzo) and is the only Italian cheese made with pig rennet, an unusual practice dating back to Roman times [18]. Lamb and calf paste are used instead in the production of PR and PS cheeses, respectively.

Because of their high commercial value, these three specialties may be subjected to frauds, for instance through the alteration of milk origin and cheesemaking process. Since cheese production is a relatively long and complex process, requiring controlled conditions and specialised know-how, cheese adulteration cannot be mimed in laboratory. To overcome such limitation, the possibility of discriminating dairy products coming from different but well documented origins and technological procedures represent an efficient way to check the robustness of a given analytical method and the suitability of the selected markers to detect deliberate and fraudulent alterations of the milk origin and cheesemaking process. The three investigated Pecorino cheeses, apart from their good reputation and appreciation by consumers, seem to be particularly suitable for this purpose, because they are all certified specialties produced in Central Italy, namely under not very dissimilar pedologic and climatic conditions. In addition, the production territories partially overlap in the case of PS and PR cheeses, which permits to evaluate whether different cheesemaking conditions applied to milk and other raw materials coming from the same geographical area can affect the mineral composition of Pecorino cheeses.

To attempt a discrimination of the above three typical Pecorino cheeses, an ICP-OES method combined to microwave-assisted digestion was developed and validated. Representative samples of the three Pecorino varieties were successively analysed and the multi-elemental data were processed by both unsupervised and supervised multivariate statistical tools. Principal Component Analysis (PCA), in particular, was at first carried out to explore the ICP-OES data matrix and the Pecorino cheese samples were finally classified according to their geographical origin/cheesemaking process by Partial Least Square-Discriminant Analysis (PLS-DA). 

## 2. Results

### 2.1. Validation of ICP-OES Analysis of Pecorino Cheese

The ICP-OES method developed for the elemental analysis of Pecorino cheese was validated according to the EURACHEM guidelines [19]. The figures of merit associated to this method are summarized in Table 1. Linearity and sensitivity were derived for each element from the related analytical calibration curve as determination coefficient (R^2^), and Limit of Detection (LOD) and quantification (LOQ), respectively. The observed R^2^ values are greater than 0.9995, except for Fe (R^2^ = 0.9921), ensuring the good linearity for all the elements in the working range investigated. LOD and LOQ values were both determined according to the BEC (Background Equivalent Concentration) concept. The relations LOD = 3·RSD_blank_·BEC/100 and LOQ = 10·RSD_blank_·BEC/100 were used, where RSD_blank_ is the Relative Standard Deviation (n = 10) of the blank and BEC is equivalent to the concentration producing twice the intensity of the background [20]. Both LOD (from 0.05 to 5.64 μg/g_dry_) and LOQ (from 0.18 to 18.80 μg/g_dry_) are largely below the mean native levels of each target element in Pecorino, even for Fe, which presents a content in the sample five times the corresponding LOQ values. Therefore, sensitivity is good enough to ensure a reliable determination of the detected elements in the real samples. Accuracy was evaluated by a spike recovery analysis because a Certified Reference Material for cheese matrix was not available. Six genuine samples and six fortified ones were analysed (see details in Section 4.3) to estimate the mean recoveries (R (%)) of the target elements (Table 1). Precision of the ICP-OES method was estimated by the Relative Standard Deviation (RSD (%)) determined in six procedural replicates. Comparison of the above two parameters with the acceptable values reported in the specialised literature [21,22] confirms that the eight detected elements can be determined with satisfactory accuracy and precision. As reported in Table 1 indeed, the elements Ba, Fe and Zn (1–50 μg/g range of concentrations) showed R(%) values from 83 to 102% (recovery benchmark: 80−110%) and RSD% values from 1 to 12% (AOAC threshold 7.3–11%, Horwitz threshold 11.3–16%); K and Mg (0.6 μg/g–2 mg/g range of concentrations) were detected with mean recovery percentages of 98 and 99% (recovery benchmark: 90−107%) and RSD% values from 1 to 2% (AOAC threshold 5.3%, Horwitz threshold 8%) and the elements P, Ca, and Na (8−20 mg/g range of concentrations) with mean recovery of 99−100% (recovery benchmark: 97−103%) and RSD% values from 1 to 2% (AOAC threshold 2.7%, Horwitz threshold 4%).

### 2.2. Exploratory Analysis of ICP-OES Data

Table 2 displays the mean concentrations (referred to the sample dry weight) of the eight detected elements in the three Pecorino cheese varieties along with the related standard deviations. Analysis of Variance (ANOVA), carried out to assess which means differ significantly, reveals that the mean content of Ca, Mg and P is not significantly affected by the cheese origin, whereas, on the opposite side, the mean concentrations of Ba, K and Na exhibit highly significant differences within the three groups of Pecorino cheeses. Successive pairwise comparison by least significant difference (LSD) test reveals that Ba is the only element that exhibits a significantly different mean concentration among the three Pecorino varieties, while the mean contents of other elements (Fe, K, Na, and Zn) are significantly different only between specific Pecorino pairs (Table 2). 

**Figure 2:**
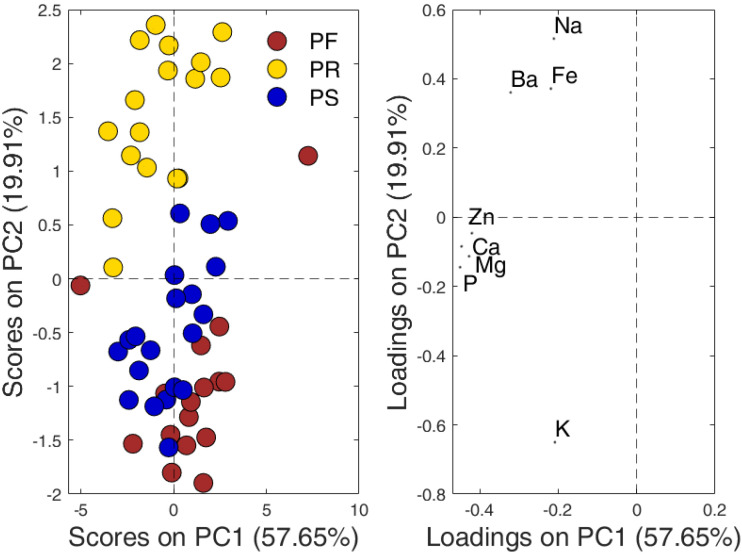
Projection of Pecorino samples (**left**) and variable loadings (**right**) on the first two principal components (PC1 and PC2).

The results of PCA exploration, performed on the auto-scaled data matrix, are displayed in Figure 2 showing the projection of both samples and variables in the plane of the first two PCs. It can be observed that the samples belonging to each of the three Pecorino varieties are distributed approximately along the bisector of the graph. Therefore, this direction describes the variability internal to each kind of Pecorino that is expected to take origin from the diversity in the ripening time, cheesemaking process and location of the production site within the deputed territory. In this regard, the variability of PF samples along this direction is comparable to that of the other two varieties, despite the first cheese is produced in a relatively small territory rather than on a regional (PS) or extra-regional (PR) scale. PF is also produced in a reduced number of small dairies by small-scale cheesemaking processes generally carried out manually. This, from one side, confers unicity to the product, as recognized by Slow Food Presidia, but, on the other, may increase the inner-class variability. PS and PR cheeses, by contrast, are often produced in medium-sized factories with the support of mechanical tools, that in compliance with the PDO specifications, ensures a better control of the cheesemaking process and, therefore, should reduce dissimilarity among different cheese lots.

The Pecorino samples grouped according to the origin along PC2, although some of the three subgroups partially overlap. The PR samples, all having positive PC2 scores, are separated from PS and PF individuals, which fall at negative values of this component and whose discrimination in the PC1-PC2 plane is incomplete. The investigation of the loadings makes apparent the sequential location of PF, PS, and PR clusters along PC2 is mainly associated to the varying content of Na, Ba, Fe, and K (a more detailed interpretation of the loading plot is provided in Section 3). 

### 2.3. PLS-DA Geographical Classification of Pecorino Samples

**Figure 3:**
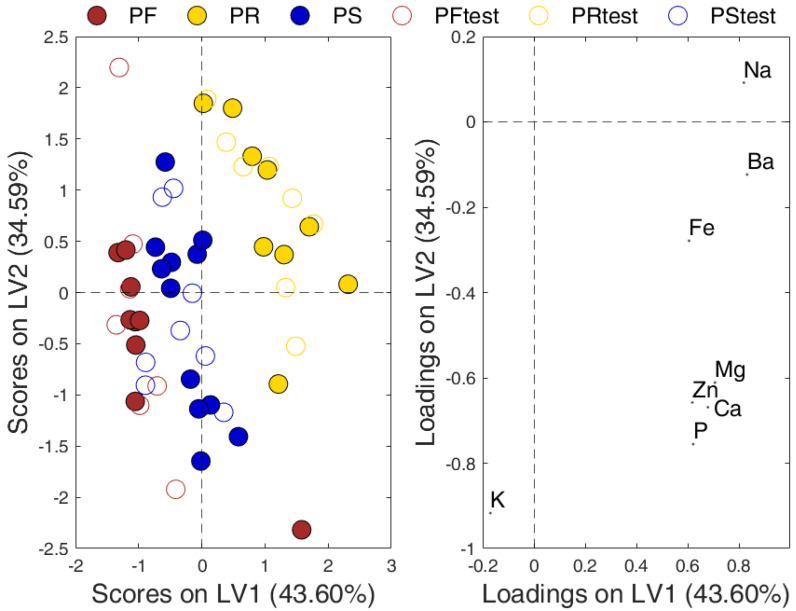
Projection of Pecorino samples (**left**) and variable loadings (**right**) on the first two latent variables (LV1 and LV2). Full and open circles identify the training and the test samples, respectively.

Prior to the creation of the classification model, the Duplex algorithm [23] was applied on each class separately to divide the samples into a training and a test set. Of the 53 Pecorino cheese samples, 30 (9 PF, 12 PS and 9 PR) were selected as the training set, while the remaining 23 (7 PF, 8 PS and 8 PR) were left out for the external validation of the model (test set). PLS-DA was optimized applying Linear Discriminant Analysis (LDA) on the calculated/predicted-Y responses in a 5-fold cross-validation procedure and exploring the evolution of classification error by increasing the number of latent variables. The classifier was tested on raw- and logarithmic scaled matrix, but no classification improvement was observed after pre-processing of the data; regardless the pretreatment used, data was auto-scaled prior to calculations. The PLS-DA model with three latent variables, which explained 85% of variance on X-block and 95% on Y-block, was eventually retained. This model performed very well on the training set, showing 93.3% accuracy in cross-validation, corresponding to the misclassification of 1 PF and 1 PR sample. A similar accuracy was observed in prediction (91.3%) proving a great stability and balance between the training and test set. All the external samples belonging to PF and PR classes were correctly assigned, while only two PS samples were misclassified. A graphical representation of the results of the PLS-DA analysis is provided in Figure 3. A further inspection of the Variable Importance Projection (VIP) [24] scores allowed the identification of the variables contributing the most to the model, according to the “greater-than-one” criterion. VIP analysis identified only three significant predictors, i.e., the elements Ba, K and Na. Afterwards, a novel PLS-DA model was built on the reduced data set (i.e., exploiting only Ba, K and Na); nevertheless, the classification performance provided by this further model was the same as the complete model. The variable selection only reduced the intra-class variances in the space of the latent variables, in line with the considerations reported in Section 2.2.

## 3. Discussion

Looking at the PCA score and loading plots displayed in Figure 2, it can be highlighted that PC1 seems to describe the overall mineral content in the cheeses, whereas the sequential location of PF, PS, and PR samples along PC2 is mainly associated to the varying content of K (negative loadings on this component) and Na, Ba, and Fe group (all having positive loadings). Such behaviour cannot be interpreted in a straightforward way because information about the processing-variables, potentially affecting the mineral composition of the final product, is not always explicitly reported in the production guidelines. Nevertheless, the results here obtained seems to be consistent with those of a previous investigation dealing with the role of raw milk source, type of rennet, salting-procedure and ripening time on the mineral composition [25]. In general, cheeses produced with sheep’s milk [26], higher ripening time and the use of animal rennet [27] present an higher overall content of minerals. Moreover, the type of rennet, that influences moisture, pH, fat and protein content [28], is expected to affect also the mineral composition. Concerning salting procedure, PR and PS are produced using both immersion (wet) and sprinkling (dry) salting, whereas only dry-salting is applied in PF production. It is reported that immersion in a brine bath is the technique resulting in the lowest level of potassium [25], in agreement with the negative scores of most of the PF samples on PC2. On the contrary, the level of Fe is reported to be higher in wet-salted cheeses, which agrees with the observed anti-correlation of K and Fe variables. Na contents are higher in longer ripening time cheeses, which justifies the location of PR, commonly known as a “salty” cheese, at positive PC2 scores. Multi-elemental composition is also affected by many other factors, i.e., animal breeding [29], physiological and environmental conditions of the animals [30], and pH value during cheese processing (influencing mineral losses), but these effects cannot be invoked here because the above conditions are not described in the production guidelines of the investigated cheeses. 

As previously discussed, the variability in the multi-elemental composition within the three Pecorino classes can be also affected by the fact that the cheeses are produced in two different manufacturing cycles and, in the case of the PS and PF groups, at two different levels of ripening. Although not reported in Figure 2 for clarity, the PS samples belonging to the “dolce” category are mainly characterised by higher scores on PC2, whereas discrimination of the PF samples according to the ripening time was less evident. Concerning the effect of the manufacturing cycle, no differentiation of the PS samples was observed, while a clear and a less perceptible differentiation of PR and PF samples, respectively, was detected in the PC1-PC3 plane. 

The same three varieties of Pecorino cheese were previously classified by PLS-DA using the volatile profiles [1], which is worth comparing to the present study. The PLS-DA model described in Section 2.3 exhibits a good prediction ability, being capable of correctly classifying 91% of the external samples (21 out of 23), despite only few elements (K, Na and Ba) are significant. As displayed by ANOVA and LSD tests reported in Table 2, higher Na and lower K contents are typical of PR cheese, while a significant increase of Ba concentration can be observed in the three Pecorino varieties following the order PS < PF < PR. The predictive performance of the PLS-DA model based on the volatile profiles, although built on 14 components of the cheese aroma, was not as good as that provided by the above three significant elements. Moreover, interpretation of the PLS-DA model based on the volatiles is particularly difficult because of the many variables affecting the complex process of cheese aroma generation. Preliminary exploratory PCA analysis conducted on the volatile profiles revealed a separation of PF samples and a partial overlapping of PS and PR individuals. This suggests that geographical origin of milk has a relevant role in the differentiation of the three varieties according to the aroma, whereas the different mineral composition of the three varieties is also dependent on cheese-making technology, such as the salting mode.

## 4. Materials and Methods

### 4.1. Pecorino Cheese Samples

The PD and PR cheese samples, all exhibiting the PDO mark, were purchased from local supermarkets while PF samples were provided by consortium “Pecorino di Farindola”, which guarantee the authenticity of all the analysed products. Fifty-three (53) samples were finally available for the discrimination study (16 PF, 20 PS and 17 PR). The samples were collected from September 2018 to June 2019, and, consequently, they belong to, at least, two different manufacturing cycles. Additionally, we took care of representing the different ripening times according to the cheesemaking regulations: both soft- and hard-ripening PF (time of maturation from a minimum of 40 days to over a year) and PS (from 20 to 60 days for PS designated as dolce, not less than two months for PS maturo) cheese samples were collected, whereas only PR for grating (from 5 to 8 months) was available in the market, according to the PDO specifications.

### 4.2. Chemicals

A multi-element TraceCERT^®^ standard solution for ICP (Fluka Analytical, Sigma Aldrich) containing Ba (at 40 mg/L), Fe and Zn (both at 100 mg/L) and mono-element TraceCERT^®^ certified standards for AAS of Ca, K, Mg, Na and P (1000 mg/L in nitric acid), purchased from Sigma Aldrich, were used to prepare an analytical calibration curve with six standard solutions (by dilution in 50 and 100 mL polymethylpentene volumetric flasks) plus a blank consisting of ultrapure water (18.2 MΩ cm resistivity at 25 °C) obtained with a water purification system MilliQ (Millipore, Germany). Hydrogen peroxide solution (≥ 30% *w*/*w*) for ultra-trace analysis from Sigma-Aldrich (St. Louis, MO, USA) and suprapure nitric acid (65% *w*/*w*) from Merck KGaA (Darmstadt, Germany) were used to digest the samples.

### 4.3. Sample Preparation and Microwave-Assisted Digestion

Pecorino cheese samples were dried in a Büchi TO-50 (Büchi Labortechnik AG, Flawil, Switzerland) drying glass oven at 75 °C for 24 h under moderate vacuum and successively ground in a mortar (cleaned after each sample preparation with an aqueous solution at 2% *v*/*v* of HNO_3_ and ultrapure water) before the digestion procedure. Aliquots of 0.3 g of cheese were transferred into teflon vessels and, after the addition of 5 mL of hydrogen peroxide and 5 mL of nitric acid, were digested in an Ethos One (Milestone, Bergamo, Italy) microwave oven at constant power (1000 W). Complete mineralization was obtained in 40 min at the constant temperature of 180 °C (reached in 10 min). After cooling at room temperature, the obtained solutions were diluted with ultrapure water into 25 mL PMP volumetric flasks. Fortified samples were prepared in the same way to check the trueness of the analytical method, in the absence of a Certified Reference Material for this matrix. Aliquots intended to serve as both genuine and enriched samples were collected by the same slice of cheese, accurately homogenised. Recovery studies were performed separately for micro- (Ba, Fe and Zn) and macro-elements (Ca, Na, P, K and Mg) taking into account of the sample mass (0.3 g) and the different dilutions used in the measurements. The spiking levels were chosen according to native concentrations of the detected elements in Pecorino cheese (determined in preliminary analyses) i.e., 2.67 μg/g_dry_ of Ba, 6.67 μg/g_dry_ of Fe and Zn (dilution to 25 mL) and 83.3 μg/g_dry_ of Mg, 166.7 μg/g_dry_ of K, 833.3 μg/g_dry_ of Na and P, and 1666.7 μg/g_dry_ of Ca (dilution to 100 mL).

### 4.4. ICP-OES Analysis

The digested samples were transferred into polypropylene vials for the simultaneous analysis of eight elements (Ba, Ca, Fe, K, Mg, Na, P and Zn) by means of an Iris Intrepid ER/S Thermo-Elemental (ThermoScientific, Waltham, MA, USA) ICP-OES spectrometer equipped with an Echelle grating optical system and a charge injection device (CID) solid-state detector. A Timberline II (ThermoScientific) autosampler collected the samples with a flow rate of 1.85 mL/min, controlled by a peristaltic pump. Each sample was conveyed in a concentric pneumatic nebulizer, where it was nebulized (nebulizer gas flow of 0.6 mL/min) into the argon plasma, connected to a cyclonic spray chamber and eventually analysed in radial torch-reading configuration using the operating conditions recommended by the manufacturer, i.e., radiofrequency power of 1.202 kW, coolant gas flow of 12 L min^−1^ and auxiliary gas flow of 0.5 L min^−1^. Ultra-High Purity 5.0 Grade Argon (99.999% pure argon) was used in the spectrometric analysis. The emission lines that provided the maximum signal to noise ratio and minimum spectral interferences were selected and a manual background correction of the emission intensity was performed. Each measurement, both on standards and digested samples, was performed in four replicates and the mean value was taken. Matrix effects, possible non-spectral interferences and instrumentation drift were monitored using a 200 µg/L Yttrium solution as Internal Standard (line 324.228 nm). Cleanliness of the introduction system and absence of memory effects were controlled by the analysis of one standard solution (0.024 μg/mL for Ba, 0.06 μg/mL for Fe and Zn, 10 μg/mL for Mg, 20 μg/mL for K, and 30 μg/mL for Ca, Na and P) followed by a blank every six samples. The Pecorino samples were analyzed in a random order.

### 4.5. Multivariate Statistical Analysis

Principal Component Analysis (PCA) was preliminarily performed to assess the similarity/dissimilarity in the multi-elemental composition within the Pecorino samples. PCA [31] allows to represent multivariate data in a low-dimensionality space of mutually orthogonal, thus uncorrelated, principal components (PCs). They can be defined as the linear combination of original variables explaining unrelated portions of information. Transformation of the original data matrix **X** is described by the Equation (1):

**X** = **TP**^T^ + **E** (1)

The loading matrix **P** (with dimension V × A, where V are the original variables and A the number of principal components) defines the new directions. The scores matrix **T** (S × A, where S is the number of samples and A the number of principal components) expresses the coordinates of the samples in the PC space. The error matrix **E** (S × V,) collects the residuals associated with the approximation of the original data with fewer PCs than the original variables. To display multivariate information, objects and loadings can be projected onto the compressed PC subspace; this provides a graphical and straightforward visualisation of the trends within the data samples (score plot) and interpretation of the selected PCs in terms of the original variables (loading plot). For exploratory analysis, visualisation of the data distribution by considering the scores and loadings plot of just the first components (generally two or three) is informative enough, because loss of useful information is generally negligible.

In the present work, Partial Least Squares Discriminant Analysis (PLS-DA) [32,33] was used as discriminant classifier. This approach has been developed as a direct extension of the Linear Discriminant Analysis (LDA) [34] and it was conceived to overcome the issues associated to the non-invertibility of the variance–covariance matrix.

PLS-DA is based on the possibility of transforming a classification problem into a regression one thanks to the mediation of a *dummy Y* response codifying the class-membership [35]. Basically, each individual can be associated to a binary y-vector encoding the class-information. For instance, for a three-category case, samples belonging to class A, class B, and class C will be identified by the vectors $y_{A}=\left[1\;0\;0\right]$, $y_{B}=\left[0\;1\;0\right]$, and $y_{C}=\left[0\;0\;1\;\right]$, respectively. This allows the creation (and the subsequent solution) of a classification problem solvable by means of PLS. Once the calibration model is built and the regression coefficients estimated, new samples can be classified. The application of the model on a novel set of observations provides a continuous, non-categorical, $\hat{Y}$. The association of novel samples to the different classes can be carried out in different ways. In the present work, the Bayesian solution proposed by Perez et al. [36] has been used. PCA and PLS-DA were performed using in-house routines in the MATLAB environment (R2020b; The Mathworks, Natick, MA, USA).

## 5. Conclusions

From the inspection of the outcomes of the PCA and PLS-DA models illustrated in the previous sections, it is quite evident the diverse classes of Pecorino present noticeable differences among one another. As expected, the divergencies initially highlighted by the PCA were confirmed by the PLS-DA model. As described, these discrepancies are not based solely on the diverse origins of the cheeses, but also on the different procedures followed for their preparation. The elemental analysis allowed seeing macroscopic differences among the concentrations of the 8 investigated elements; nevertheless, the VIP analysis opened up to a more refined interpretation of which variables contribute the most to the classification model. In particular, in complete agreement with the outcome of the ANOVA, it became apparent the discrimination is mainly due to Ba, Na, and K. The inspection of the PCA-loadings plot revealed that, of these, the first two are found at higher concentrations in PR samples than in the other two classes; on the contrary, K is particularly high in PS and PF, whereas is anticorrelated with PR.

As far as the predictive aspect of the classification model is concerned, it is evident that the PLS-DA model is robust and reliable, and it erroneously classifies only two test samples, belonging to class PS. A more in-depth investigation of these individuals has shown that they are both Pecorino *dolce*, i.e., soft-ripening; this aspect surely influenced their mineral composition and, consequently, their class-assignment.

## Figures and Tables

**Table 1:** Working linear range (WLR), determination coefficient (R^2^) and limit of detection (LOD) and quantification (LOQ) related to the analytical calibration curve of the elements (with corresponding detection wavelength, λ) determined by ICP-OES. Mean recovery (R) and relative standard deviation (RSD) observed in the analysis of the enriched samples (n = 6).

Element	λ (nm)	R^2^	WLR (µg/mL)	LOD (µg/g_dry_)	LOQ (µg/g_dry_)	R(%)	RSD (%)
Ba	233.527	0.9998	0.008–0.08	0.05	0.18	97	7
Ca	315.887	0.9997	10–100	3.38	11.27	99	2
Fe	259.940	0.9921	0.02–0.20	0.22	0.73	83	12
K	766.491	0.9996	5–50	1.43	4.78	98	1
Mg	280.271	0.9997	1–30	1.01	3.35	99	2
Na	588.995	0.9996	10–100	5.64	18.80	99	1
P	213.618	0.9998	10–100	2.80	9.33	100	2
Zn	202.548	0.9975	0.02–0.20	0.24	0.81	102	1

**Table 2:** Mean concentrations and related standard deviations of the detected elements in the Pecorino cheese samples. Significance (*p*-value) of difference in the means determined by one-way ANOVA and list of the significantly different class pairs according to LSD test.

Element	PF (n = 16)	PS (n = 20)	PR (n = 17)	ANOVA (*p* Value)	LSD ^§^
Ba *	1.2 ± 0.7	2.7 ± 0.8	3.5 ± 1.1	<10^−4^	PF-PS; PS-PR; PF-PR
Ca ^$^	12 ± 3	13 ± 2	13 ± 3	0.3070	-
Fe *	2.4 ± 1.7	2.5 ± 1.3	3.8 ± 1.8	0.0283	PF-PR; PS-PR
K ^$^	1.7 ± 0.3	1.5 ± 0.4	1.1 ± 0.3	<10^−4^	PF-PR; PS-PR
Mg ^$^	0.65 ± 0.19	0.70 ± 0.12	0.73 ± 0.16	0.3331	-
Na ^$^	11 ± 5	10 ± 2	21 ± 5	<10^−4^	PF-PR; PS-PR
P ^$^	8 ± 2	8 ± 2	9.0 ± 1.7	0.6910	-
Zn *	40 ± 12	48 ± 11	49 ± 15	0.0711	PF-PS; PF-PR

^$^ mg/g_dry_; * µg/g_dry_; ^§^ 0.05 significance level.

## Data Availability

Not Applicable.
